# Novel 3D-Printed Cell Culture Inserts for Air–Liquid Interface Cell Culture

**DOI:** 10.3390/life12081216

**Published:** 2022-08-10

**Authors:** Magdalena Bauer, Magdalena Metzger, Marvin Corea, Barbara Schädl, Johannes Grillari, Peter Dungel

**Affiliations:** 1 Ludwig Boltzmann Institute for Traumatology, The Research Center in Cooperation with AUVA, 1200 Vienna, Austria; 2 Austrian Cluster for Tissue Regeneration, 1200 Vienna, Austria; 3 University Clinic of Dentistry, Medical University of Vienna, 1090 Vienna, Austria; 4 Institute of Molecular Biotechnology, University of Natural Resources and Life Sciences, 1180 Vienna, Austria

**Keywords:** tissue engineering, 3D printing, PLA, full-thickness skin model, insert

## Abstract

In skin research, widely used in vitro 2D monolayer models do not sufficiently mimic physiological properties. To replace, reduce, and refine animal experimentation in the spirit of ‘3Rs’, new approaches such as 3D skin equivalents (SE) are needed to close the in vitro/in vivo gap. Cell culture inserts to culture SE are commercially available, however, these inserts are expensive and of limited versatility regarding experimental settings. This study aimed to design novel cell culture inserts fabricated on commercially available 3D printers for the generation of full-thickness SE. A computer-aided design model was realized by extrusion-based 3D printing of polylactic acid filaments (PLA). Improvements in the design of the inserts for easier and more efficient handling were confirmed in cell culture experiments. Cytotoxic effects of the final product were excluded by testing the inserts in accordance with ISO-norm procedures. The final versions of the inserts were tested to generate skin-like 3D scaffolds cultured at an air–liquid interface. Stratification of the epidermal component was demonstrated by histological analyses. In conclusion, here we demonstrate a fast and cost-effective method for 3D-printed inserts suitable for the generation of 3D cell cultures. The system can be set-up with common 3D printers and allows high flexibility for generating customer-tailored cell culture plastics.

## 1. Introduction

Human skin models are important tools for research, clinical purposes, and industrial applications. Animal models are often used in the area of skin research [1], as the 2D monolayer of in vitro models lacks cell–cell and cell–matrix interactions and does not exhibit many physiological behaviours. Monolayer cultures allow only very limited inference about the physiological responses of organisms to external stimuli. In vivo animal models can provide that complex information, however, there has been a general concern for the animal suffering experienced during research. This is what inspired W. Russell and R. Burch to write and publish a guide to maximally reduce animal pain in 1959 [2]. There, they established the principles of the 3Rs: replacement, reduction, and refinement. Today the 3Rs are fixed in legislation regulating the use of animals for scientific studies, such as Directive 2010/63/EU [3] for the European Union. The attempt to close the in vitro–in vivo gap necessitates novel in vitro model systems that closely mimic the human skin and forecast the physiological behaviour of healthy and diseased skin tissue.

The skin forms the protective layer of the body which separates the organism from the environment. With a total area of around 1.7 m^2^ and weighing in at 16% of the body mass, the skin is the largest and one of the most complex human organs. Depending on functionality, structure, and cell type location, the skin is divided into three layers (epidermis, dermis, and hypodermis), which are again subdivided into distinctive layers. The epidermis contains mostly keratinocytes, which are epidermal cells. However, there are also melanocytes, lymphocytes, Langerhans cells, and Merkel cells in this layer. The majority of cells in the dermis are fibroblasts, which are dermal muscle cells. Migratory immune cells, such as leukocytes and lymphocytes are also present in this layer. The hypodermis is loose connective tissue with a large proportion of adipocytes [4]. To engineer these complex multi-layered, multicellular anatomic structures is technically challenging. In addition to promotion of epidermal differentiation with supplements (e.g., the calcium switch, triggered by high calcium conditions [5]), the modelled skin tissue is raised to the air–liquid interface (ALI) to mimic physiological conditions. In contrast to ordinary immersed cultures, cells are grown on a basement membrane. The membrane can be raised to the air–medium interface after eligible culture periods, resulting in exposure to air on one side of the culture [6]. Besides skin equivalent cultures, the technique is conventionally used to culture airway epithelial cells [7] and organoids [8,9]. For the modelling of skin equivalents, this technique is the gold standard.

Depending on the scientific question, skin equivalents can be modelled in different ways. With each layer and each additional cell type, a level of complexity is added. To mimic the dermis (dermal equivalent), fibroblasts are either seeded in multilayers [10] or onto a chosen matrix (such as de-epidermised dermis, hydrogel, or scaffold material). The epidermis, with its distinctive layers, is more complex to create. Epidermal equivalents must be modelled under delicate conditions to achieve stratification and cornification [11]. The chosen method involves the initial seeding of keratinocytes onto the dermal model. The result of this process is called full-thickness skin equivalent (FTSE). These equivalents offer high-end complexity and provide physiological behaviour regarding the interplay of fibroblasts and keratinocytes, resulting in a plethora of use cases for modelling cancer; aging [12], or long-term toxicology experiments [13].

Ready to use FTSEs are commercially available from companies, such as T-skin™ (Episkin, L’Oréal, Lyon, France), EpiDerm™ (MatTek, Ashland, MA, USA), or Phenion^®^ (Henkel AG & Co. KGaA, Düsseldorf, Germany). These products are standardized and quality-controlled but expensive. To overcome the cost issue, SEs are also laboratory-made using commercially available plastic ware. The plastic ware, however, is still costly and lacks variability for specific downstream applications, allowing only applications similar to commercially available products. Recently, 3D printing technology has been introduced to the biomedical engineering and cell culture field. This technology has several advantages compared to conventional manufacturing processes, such as increased versatility, cost-effectiveness, and higher efficiency. The freedom of design allows high flexibility in the generation of new setups for cell culture [14]. Thus, the present study aimed to design and develop novel cell culture inserts based on 3D printing technology, optimized for the application of the ALI method. The feasibility of these inserts was tested by following a basic protocol to generate skin-like tissues.

## 2. Materials and Methods

### 2.1. Construction and Printing of Cell Culture Insert

**Figure 1:**
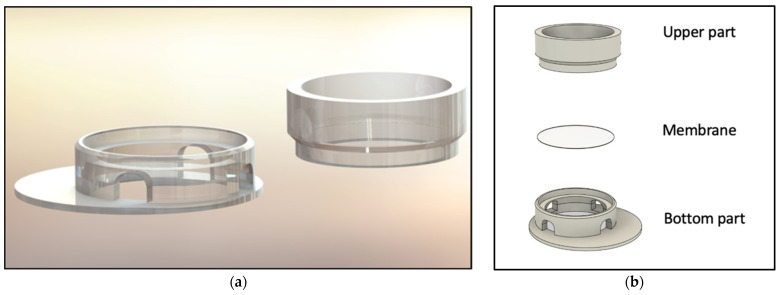
Visualization of the 3D-printed cell culture insert. (**a**) Rendered insert construction. (**b**) Schematic of the device with upper and lower parts of the inserts. The customized membrane is indicated in between.

**Figure 2:**
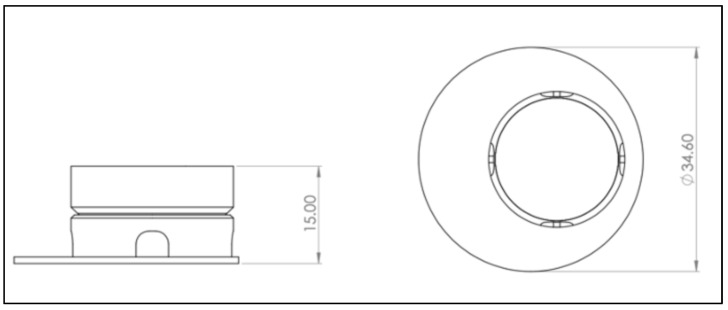
Drawing derivation of computer-aided design (CAD) model of the 3D-printed cell culture insert. Left: Front view. Right: Bird’s eye view. A more detailed plan can be found in Appendix B Figure A2, Figure A3, Figure A4 and Figure A5. Scale in mm.

The computer-aided design model was constructed using Solidworks 2021 software (Dassault Systèmes, Vélizy-Villacoublay, France). The final construction is visualized in Figure 1 and Figure 2. For printing, an Ender 3 Pro (Shenzhen Creality 3D Technology, Shenzhen, China) printer was utilized. All inserts were printed with polylactic acid (PLA) (Shenzhen Getech Technology, Shenzhen, China) of transparent colour. Printing parameters were tested by trial and error, changing fill density, printing temperature, printing speed, retraction, and retraction speed. The final printing parameters are shown in Table 1. To create the rendered graphics of the constructs, Solidworks PhotoView 360 software (Dassault Systèmes, Vélizy-Villacoublay, France) was used. The construction templates of the upper (Supplementary Materials S1) and lower part (Supplementary Materials S2) of the inserts as well as an animation video (Supplementary Materials Video S1) can be found via the link given below. 

To sterilize the 3D-printed inserts for cell culture use, the inserts were first submerged in 70% ethanol for at least 15 min. Then, the inserts were assembled by putting a Nucleopore Track-Etch membrane (Whatman, Maidstone, UK) with 3 µm pores between the lower part and the upper section of the device. Subsequently, the assembled insert was irradiated in the laminar flow for at least 15 min with ultraviolet (UV) light. After the irradiation, the inserts were placed into 6-well plates (CoStar Group, Washington, DC, USA) for further use.

### 2.2. 3D Cell Culture

For generation of the 3D co-cultures, a human dermal fibroblast cell line (fHDF/TERT166, Evercyte, Vienna, Austria) and normal human epidermal skin keratinocytes (NHEK/SVTERT 3-5, Evercyte, Austria) were used. Basic cell culture was conducted according to the manufacturer’s instructions. Cells were cultured at 37 °C, 5% CO_2_, and moderate humidity. Thawing of the fibroblasts and keratinocytes was conducted according to manufacturer’s instructions.

Expansion of fibroblasts was performed in T175 flasks (Greiner Bio-One, Kremsmünster, Austria) with a 1:1 medium mixture of Dulbecco’s Modified Eagle Medium (DMEM-high glucose containing phenol red, Sigma-Aldrich, St. Louis, MO, USA) and Ham’s F-12 (Lonza, Basel, Switzerland) supplemented with 10% FCS (Sigma-Aldrich, USA), 2 mM glutamine (GlutaMax, Gibco, New York, NY, USA) and 1 µg/mL G148 (InvitroGen, Waltham, MA, USA). When cells reached a confluency over 80–90%, they were split in a ratio of 1:3 or 1:4. Cells were washed twice with PBS (Lonza, Basel, Switzerland) before adding 2.5× Trypsin/EDTA (Sigma-Aldrich, USA) diluted 1:4 in PBS for 5 min at 37 °C. The reaction was stopped by adding double the amount of cell culture medium. Expansion of keratinocytes was also performed in T175 flasks with KBM-2 growth medium (Lonza, Basel, Switzerland), supplemented with KGM-2 keratinocyte medium SingleQuot kit (Lonza, Basel, Switzerland). To detach the cells, a 1:2 dilution of 2.5× Trypsin/EDTA in PBS was used after two washing steps with PBS. The reaction was stopped with equal the amount of trypsin neutralizing solution (Gibco, New York, NY, USA) and washed with 10 mL PBS. From this step onwards, the procedure was identical for both cell types. The cell suspensions were centrifuged at 100× *g* for 5 min in a 50 mL Falcon Tube (Greiner Bio-One, Kremsmünster, Austria). Cell pellets were resuspended in fresh medium and seeded into a T175 flask. In the case of subconfluency, cell culture medium was exchanged every third or fourth day.

To model the dermis, a collagen/fibrin hydrogel mix was prepared and subsequently loaded with fibroblasts. For this purpose, a fibroblast suspension was prepared in fetal calf serum (FCS) (Sigma-Aldrich, St. Louis, MO, USA) with an end concentration of 1 × 10^5^ cells/mL. Mixing of the gel was conducted on ice to prevent premature clotting. After preparing the cell suspension, Hank’s Balanced Salt Solution (HBSS) with phenol red (Gibco, New York, NY, USA) was added dropwise to collagen (Collagen G, type 1, solution 4 mg/mL (Sigma-Aldrich, St. Louis, MO, USA)). The pH was slowly adjusted to approximately 7.5 with 1 M sodium hydroxide solution until the mixture turned red/pink. After adjusting the pH, the fibroblast/FCS solution was added to the mixture. The proportions were one-part HBSS, one part fibroblast/FCS suspension, and eight parts collagen. Before adding the cell suspension to the collagen, thrombin (TISSEEL Lyo, Baxter, Deerfield, IL, USA) was added to the fibroblast/FCS suspension to an end concentration of 1 U/mL. To start the gelling, fibrinogen, dissolved in aprotinin, was added to the final mixture to an end concentration of 3 mg/mL. Next, 2500 μL of collagen/fibrin gel containing fibroblasts was quickly transferred without air bubbles into each insert. Plates were incubated at 37 °C, 95% humidity, with 5% CO_2_ until the collagen/fibrin matrix was solid. To equilibrate the gel, 1000 μL keratinocyte growth medium was pipetted into each insert and the outer well was filled above the membrane level (appr. 4.5 mL). Equilibration for at least one hour was conducted in the incubator. Next, for the epidermal layer, keratinocytes were detached according to the standard cell culture procedure. A cell suspension of 1.5 × 10^6^ cells/mL was prepared in keratinocyte culture medium. After the removal of the equilibration medium from the inner insert, 1000 μL of keratinocyte suspension was added on top of the solid gel. The SEs were then incubated overnight at 37 °C, 5% CO_2_, and moderate humidity. The next day (Day 2), the SEs were loosened by moving a 200 μL pipette tip carefully around the outer wall of the insert to avoid tension stress during the culturing. On Day 4, the medium was changed from keratinocyte growth medium to differentiation medium. For that, 500 mL KGM medium (Lonza, Basel, Switzerland) was supplemented with 25 mg ascorbic acid (Sigma-Aldrich, USA), 500 mg albumin from bovine serum (BSA) (Sigma-Aldrich, USA), 5 mg transferrin (Sigma-Aldrich, USA) and CaCl_2_ (Sigma-Aldrich, USA) to an end concentration of 1.3 mM. The dry components were added to the KGM medium, and the mixture was subsequently filtrated through a 0.22 μm filter. Next, the KGM SingleQuot supplements (Lonza, Basel, Switzerland), except for the Bovine Pituitary Extract (BPE), were added. The differentiation medium was then added only to the outer wells to facilitate the ALI. For this, a volume of 4 mL per well was used. The medium was changed every other day.

### 2.3. Cytotoxicity Assay According to ISO 10993-5

Cytotoxic effects of the transparent PLA material on keratinocytes and fibroblasts in their respective medium were investigated following the ISO standard 10993-5. This standard offers the choice of different assays to determine cytotoxicity. Here, the thiazolyl blue tetrazolium bromide (MTT) assay and visible validation were chosen.

Briefly, keratinocytes were seeded at a cell concentration of 10^5^ cells/mL, fibroblasts at 10^5^ cells/mL into a 96-well plate to reach a confluency of 50% the next day. The first and the last columns were spared to avoid edge effects. Two columns were only filled with media for non-cell control. At 24 h after seeding, 100 µL treatment media was applied to the wells. The treatment medium was created by placing sterilized inserts for 48 ± 2 h into a 6-well plate (Corning, Glendale, CA, USA) filled with 6 mL keratinocyte or fibroblast growth medium respectively. The treatment medium was applied in concentrations ranging from 0–100%. The 0% treatment media was equal to the fresh cell culture medium, 50% was a 1:1 mixture of fresh and 100% medium, that was incubated with the insert. Cells were incubated for 24 h with the various treatment media groups. Then, 25 mg of MTT powder (Thermo Fisher Scientific, Waltham, MA, USA) was dissolved in 5 mL sterile PBS to make a 5 mg/mL MTT stock solution. Next, the treatment medium was discarded. Then, 50 µL of MTT solution generated by diluting the stock solution in respective medium was added to each well. After an incubation period of 2 h at 37 °C, the reagent was removed from each well. Afterward, 100 µL isopropanol (Sigma-Aldrich, USA) was added, and plates were incubated for 30 min on an orbital shaker (VWR International, Radnor, PA, USA). Absorbance was then measured at 570 nm (reference wavelength 650 nm) on a plate reader (Polarstar, BMG Labtech, Ortenberg, Germany).

### 2.4. Histology

After culturing the epidermal and dermal components of the engineered tissue submerged for three days followed by 19 days at ALI, the whole structure together with the insert membranes was transferred to a histology cassette and fixed in formaldehyde 4% aqueous solution (VWR International, USA) for 24 h. On the next day, the samples were rinsed in tap water for 1 h, followed by dehydration in 50% ethanol for 1 h. The dehydration was completed by storing the biopsy samples in 70% ethanol. After embedding the samples in paraffine, they were cut in 4 µm thin sections. These were first deparaffinized and rehydrated, H&E staining was obtained following a standard protocol. For the immunofluorescence staining, the sections were steamed in preheated 10 mM sodium citrated buffer at pH 6 for 20 min. After cooling down, the sections were rinsed in TBS-T and blocked with normal goat serum + 0.1% bovine serum albumin (in PBS) (1:60, VECS-1000, Vector Laboratories, Newark, USA; A7030-10G, Merck KGaA, Darmstadt, Germany) for 1 h at room temperature. The primary antibodies, keratin 14 (K14) (1:200, ab24525, Abcam, Cambridge, UK), keratin 10 (K10) (1:400, GPK10, Progen, Heidelberg, Germany), and vimentin (1:1000, ab24525, Abcam, UK) were diluted in the blocking reagent and incubated overnight in a humidified chamber at 4 °C. On the next day, sections were rinsed in TBS-T three times and secondary antibodies were applied. For K14 goat anti-mouse 647 (1:1000, A32728, Thermo Fisher Scientific, USA), K10 goat anti-guinea pig 488 (1:1000, A11073, Thermo Fisher Scientific, USA), and vimentin goat anti-chicken 488 (1:1000, A32931, Thermo Fisher Scientific, USA) were chosen and incubated for 1 h at room temperature. For staining the nuclei, DAPI (1:1000, D21490, Thermo Fisher Scientific, USA) was incubated for 10 min. Finally, the sections were mounted with Mowiol (0713.2, Carl Roth, Karlsruhe, Germany).

## 3. Results

### 3.1. Construction and Printing of Cell Culture Insert

**Figure A1:**
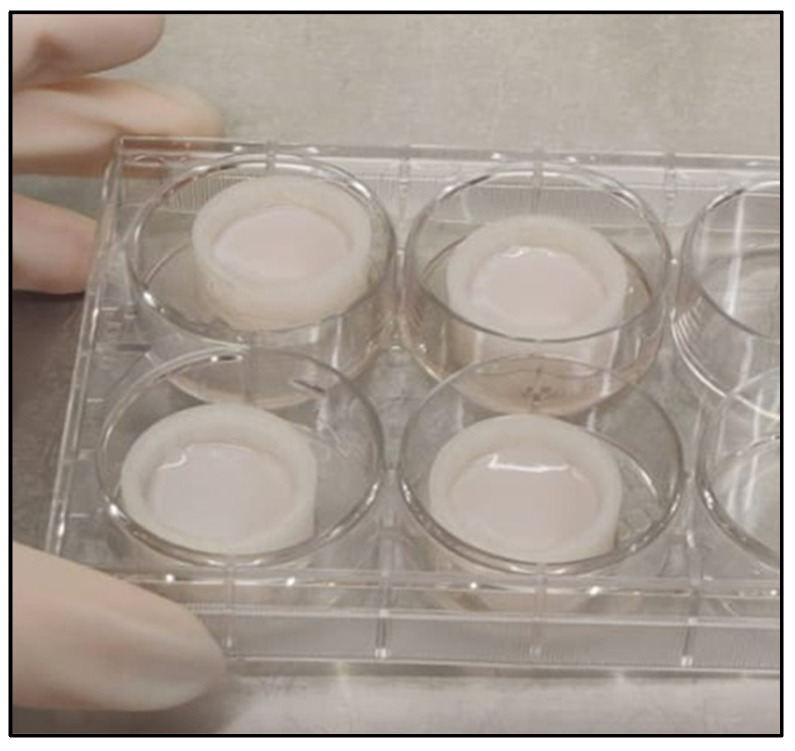
Inserts in cell culture well plate with 3D tissue on the second day after seeding.

To model an optimal 3D tissue, the main aim was to develop an insert structure optimized for liquid handling, allowing the mandatory raise of the culture to the air–liquid interface but simultaneously showing high flexibility in setup parameters. For this purpose, several versions of the device were constructed and tested in cell culture concerning functionality and handling. Regarding functionality, the following requirements were considered: sufficient space for modelling SE inside the insert, ability to exchange the membrane, compatibility with conventional well plates, as well as room for an effortless and smooth culture medium exchange. The final construction is visualized in Figure 1 and Figure 2. The SE is modelled on top of the membrane, inside the upper part, allowing the culture medium to nurture cells from the bottom side. The two-piece construction allows the membrane to be removed and replaced. To have enough space for media exchange, the insert’s cylinder is decentralized. The four windows of the cylinder allow the liquid exchange from outside the cylinder to its inside. Improvements in the design of the inserts regarding localization of cylinder inside the well, window size, and membrane localization were performed resulting in the presented final version. (See Appendix A Figure A1 for a photo of inserts in cell culture setting.)

In the course of development, several printing parameters (layer height, nozzle outlet, wall thickness, fill density, printing temperature, printing plate temperature, printing speed, moving speed, retraction, retraction speed, and printing plate adhesion type) were tested to find the optimum conditions, especially to avoid stringing, warping and to create a perfect printing surface. The final parameters leading to an even print image are shown in Table 1.

### 3.2. Cytotoxicity Assay According to ISO 10993-5

**Figure 3:**
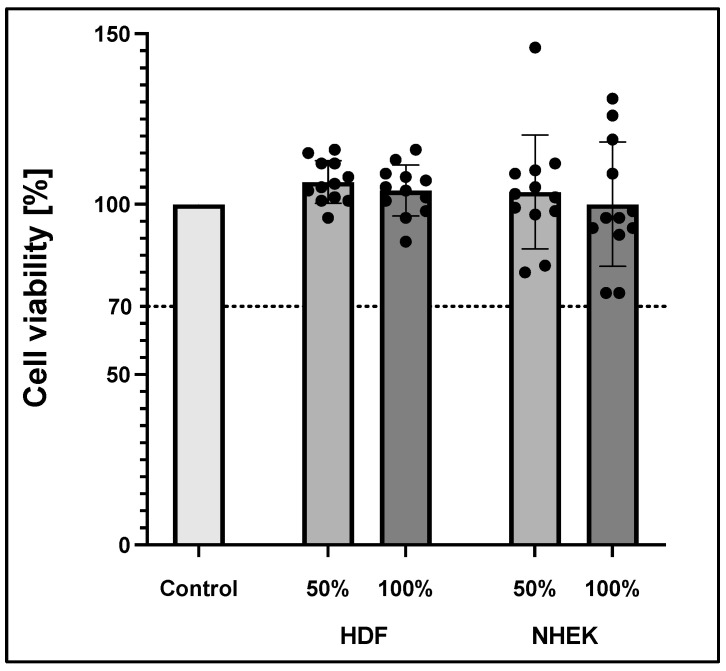
Cell viability according to ISO 10993-5 of human dermal fibroblast (HDF) and normal human epidermal skin keratinocytes (NHEK). Viability was measured via MTT assay. The values represent means ± standard deviation of two independent experiments with six technical replicates each (*n* = 12).

To exclude cytotoxic effects of the 3D-printed material, the inserts were tested according to ISO 10993-5 analysing cell viability. Briefly, inserts were incubated for 48 h in a culture medium. These medium supernatants were then withdrawn and applied to both fibroblasts and keratinocytes. Supernatants were either used undiluted (100%) or diluted 1:2 with fresh medium (50%) and compared to the effects of medium alone (0%). MTT assay was conducted after 24 h incubation time. All groups were standardized to a 0% control group, as shown in Figure 3. According to ISO 10993-5, reduced cell viability <70% indicates cytotoxic potential. Moreover, the 50% extract of the test sample should have at least the same or higher viability than the 100% extract. MTT test revealed no cytotoxic effects of the 3D-printed PLA material on both keratinocytes and fibroblast in the respective media.

### 3.3. Confirmation of Cell Culture Applicability

**Figure 4:**
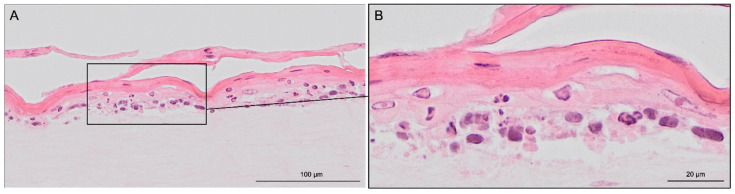
Hematoxylin & eosin staining images of skin-like co-culture modelled on 3D-printed insert. Light pink-coloured collagen-based dermis harbouring distributed fibroblasts is in the bottom part of the figure. Superior to the light pink dermis, darker epidermis with recognizable layered structures can be found. Pink stratified stratum corneum desquamate detached and elongated cells can also be seen. The tissue was generated by culturing the epidermal and dermal components submerged for three days, then allowing the epidermal compartment differentiation for 19 days at ALI. Scale bar: (**A**) 100 µm and (**B**) 20 µm.

Haematoxylin & eosin staining was used to visualize the modelled 3D structure (Figure 4). The presence of two distinct tissues was further confirmed by specific staining. The stratification of keratinocytes, indicating the formation of *stratum corneum,* was confirmed by detached and elongated cells.

**Figure 5:**
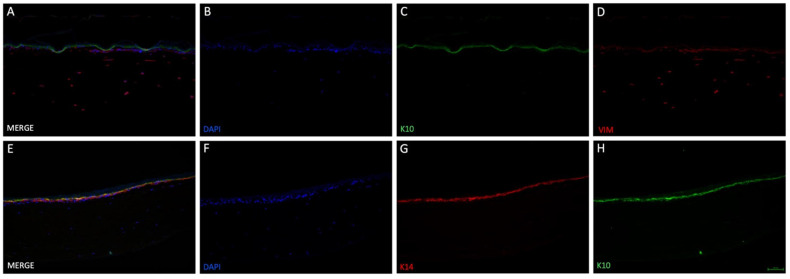
Immunofluorescence staining of two different skin-like co-cultures modelled in 3D-printed insert. 3D tissue was generated by culturing the epidermal and dermal components submerged for three days, then allowing the epidermal compartment differentiation for 19 days at ALI. The upper row (**A**–**D**) demonstrates the presence of fibroblasts in the lower compartment ((**D**), vimetin (VIM)) as well as keratinocyte differentiation ((**C**), keratin 10 (K10)). Cell nuclei were stained with DAPI (**B**) and a merged image can be seen in (**A**). In the lower row (**E**–**H**), a second 3D tissue was additionally stained with another marker for keratinocyte differentiation: keratin 14 ((**G**), K14). Furthermore, cells were positive for K10 (**H**) and DAPI (**F**). Merging all three images showed that K10 and K14 positive cells formed two layers, indicating differentiation of keratinocytes (**E**). Scale bar: 100 µm.

Vimentin, DAPI, keratin 14 and keratin 10 fluorescence staining validated the skin-like structure using the 3D-printed inserts (Figure 5). Fibroblasts could be found in the collagen-based dermis, distributed evenly through their matrix. Keratinocytes were located on top of the dermal compartment. DAPI staining revealed that the cell nuclei amount thins out upwards. *Stratum basale* appeared multi-layered in haematoxylin & eosin staining and was confirmed with keratin 14 staining. Differentiation marker keratin 10 was found superior to *stratum basale*, demonstrating the stratified nature. These results show the effectiveness of the newly designed, 3D-printed cell culture inserts.

## 4. Discussion

Commercially available cell culture inserts are cost-intensive, lack variability for specific downstream applications, and are subject to logistical problems. The present paper describes a novel, tempting laboratory-based alternative, which allows the application of the ALI method as well as easy modifications of the experimental setup. The devices can be printed fast and simply in the laboratory using extrusion-based 3D printing.

3D printing has several advantages compared to conventional manufacturing processes such as cost-effectiveness, versatility, and higher efficiency. It has impacted several biomedical engineering applications, including surgical guides, tissue regeneration, artificial scaffolds, and implants, as well as the administration and delivery of drugs. First, extrusion-based 3D printing processes require a digital computer-aided design model, which is then sliced into single layers. These layers are used to derive the path that the printer nozzle must travel to create a 3D model of the construction. This procedure allows the production of versatile 3D objects with modulable shapes and dimensions. One of the most used polymers is PLA, which was utilized in the present study [14,15].

PLA is a common material, which is the most extensively studied aliphatic polyester. It is the top 3D printing industry-leading biomaterial in replacing petrochemical-based polymers [16], derived 100% from renewable resources. PLA is highly versatile, biodegradable, biocompatible, and has suitable mechanical properties, like thermoplastic processibility [17]. The educt of PLA is lactic acid, which is a naturally occurring organic acid that can be produced by fermentation of sugars obtained from renewable resources such as sugarcane [18]. The polymer is used more and more for medical applications, like wound management [19], stent applications [20], as well as orthopaedic and fixation devices [21], tissue engineering [22], and drug delivery [23], but also finds wide use in the non-medical field [19]. Thus, PLA was the material of choice in the present study.

To realize the idea of a PLA cell culture device, a computer-aided design model was created. While improving the insert design, it went through several construction versions. Modifications after the testing of each version improved the insert from a pilot device all the way to the final operative version. The decentralized cylinder creates space for the pipette tip to enable media changes. To ensure constant media exchange from the membrane side, four windows were integrated into the cylinder. These must reach up to the membrane, otherwise, air bubbles can accumulate under the membrane, resulting in an insufficient nutrient supply. Further, enough volume space above the membrane is of importance to model the equivalents. Another point is the clamping geometry that keeps the membrane sealed between the upper and lower part. Care must be taken to achieve less clamping force, by shortening the connector area and thereby making assembly and disassembly easier. With this knowledge, the device could be realized, allowing the application of the ALI method in cell culture.

Sterile cell culture conditions are mandatory to ensure the successful modelling of SE. Autoclaving, the standard sterilization procedure, is known to deform PLA 3D-printed constructs [24,25]. Hence, we used a two-step procedure where the devices were first immersed in ethanol and then irradiated with UV light. Previous studies confirm the chemical inertness of PLA due to its lack of reactive side-chain groups [26]. This makes the material a natural choice for biomedical applications. We confirmed this knowledge with a cytotoxicity test according to ISO 10993-5, where no cytotoxic effects could be seen given our sterilization procedure and experimental conditions. Thus, the first main goal, to create optimized 3D-printed cell culture inserts, was achieved.

The subsequent second goal was to test the functionality in a cell culture setting, by modelling skin-like 3D tissue. One of the most important functions of the skin is the barrier that separates the organism from the outer environment. Without the skin’s barrier noxae, such as viruses and bacteria [27], but also physical stresses would quickly lead to the organism’s demise [28]. It prevents not only external damage but also damage caused by the loss of internal substances, e.g., the rapid loss of fluid and electrolytes in burn victims, when a significant portion of the body surface area has been destroyed [29,30]. The main physical barrier is the avascular epidermis, the skin’s most superficial layer that is stratified into sublayers. A single layer mostly made up of basal cells, which are keratinocytes, forms its base, the stratum basale. The basal cells are constantly dividing, thereby producing new keratinocytes that push the older ones further upwards, away from the stratum basale towards the stratum corneum [4]. On their way from the stratum basale to the stratum corneum, the skin cells undergo distinctive phases of development, which define the intermediary layers of the stratum spinosum and stratum granulosum. In body regions that are exposed to tear and wear, such as the palms of the hands or the soles, a fifth layer, called stratum lucidum is formed as an additional protective layer [31,32]. Underneath the epidermis, the dermis is located and consists of two layers of connective tissue, which contain blood vessels, lymphatic vessels, nerves, hair follicles, and sweat glands. The main function of the dermis is to maintain hydration of the skin. It harbours connective tissue out of an interconnected mesh of elastin and collagenous fibres produced by fibroblasts. The upper layer is the papillary layer, which is thinner, composed of loose connective tissue, and connects the epidermis with the dermis. The deeper layer is the thicker reticular layer, comprised of dense connective tissue containing collagen fibres with fewer cells in it. This layer makes up approximately 80% of the dermis [4,32].

Cell culture experiments successfully demonstrated that it is technically possible to generate 3D tissue resembling SE on the in-house 3D-printed inserts, confirmed by haematoxylin & eosin staining (Figure 4). The collagen/fibrin gel matrix did not leak in the liquid phase and stayed inside the insert before and after polymerization. Fluorescent DAPI and vimentin staining (Figure 5B,D) revealed, that fibroblasts were distributed evenly throughout the collagen/fibrin gel. Collagen is the major structural protein of most hard and soft tissues in the human body. It plays a prominent role in maintaining structural integrity, as well as the biological function of the ECM [33]. Moreover, collagen provides physical support to tissues and with around 30% of all proteins, it is the most abundant protein in mammalian bodies [34]. Fibrin and fibrinogen play a role in haemostasis and are the main factors of wound healing and several other biological functions such as thrombosis [35]. Fibrinogen is a large glycoprotein, which is cleaved by thrombin converting the soluble molecule to insoluble fibrin [36]. As fibrinogen has been shown to contain RGD integrin-binding sites which commonly bind fibroblasts [37], the acceleration and enhanced viability of fibroblasts in the present study is explicable. The tendency of an epidermis created by keratinocytes could be detected on top of the modelled dermis. Stacked over one another, differentiation markers keratin 14 (only stratum basale) and keratin 10 (stratum spinosum to corneum) could be identified using immunofluorescence staining (Figure 5C,G,H), indicating physiological tendencies of keratinocyte differentiation.

The focus of this work was the creation of functional and adjustable cell culture inserts for use in tissue engineering, which was tested by following a basic protocol for skin-like equivalents. Nonetheless, adaptions such as the addition of various factors (e.g., TGF-β, cholera toxin, ascorbic acid, aprotinin, etc.) may improve the model and yield better results, especially considering keratinocyte differentiation. An additional limitation of this study is that the immune, vascular, and lymphatic, as well as skin appendages and the nervous component of the skin, were not considered. However, as an extension to our basic model, it is possible to integrate the immune component. There are many examples in the literature in which parts of the immune system are integrated into artificial SE. The simplest method would be to add relevant cytokines but there are also approaches with incorporated Langerhans cells [38], dermal dendritic cells [39], T cells [40], and macrophages [41]. The incorporation of skin appendages and nerves is currently being investigated [42].

Consequently, the presented in-house 3D-printed insert harbours countless application options, whether it is the extension of the described generation protocol or the usage of an established protocol. The ability to remove the membrane allows for easy testing of different membrane materials. Application to other tissue types is also conceivable as two cell types can be seeded on either side of the membrane while using the ALI method.

## 5. Conclusions

The present study offers a tempting 3D-printed alternative to commercially available inserts for cell culture models at the air–liquid interface. The device was successfully established and evaluated. The system is accessible to everyone with access to a 3D printer, compatible with commonly used cell culture plates, and very cost-effective. These customer-tailored cell culture plastics using degradable PLA, additionally provide a promising green technology, compared to commercially available inserts. Researchers can choose the preferred printing material, membrane material, and therefore make various scientific questions accessible.

## Figures and Tables

**Table 1:** Final printing parameters.

Parameter	Unit	Value
Layer height	mm	0.2
Nozzle outlet	mm	0.4
Wall thickness	mm	0.8
Fill density	%	100
Printing temperature	°C	205
Printing plate temperature	°C	60
Printing speed	mm/s	50
Moving speed	mm/s	100
Retraction	mm	6.5
Retraction speed	mm/s	33
Printing plate adhesion type	/	skirt

**Figure A2:**
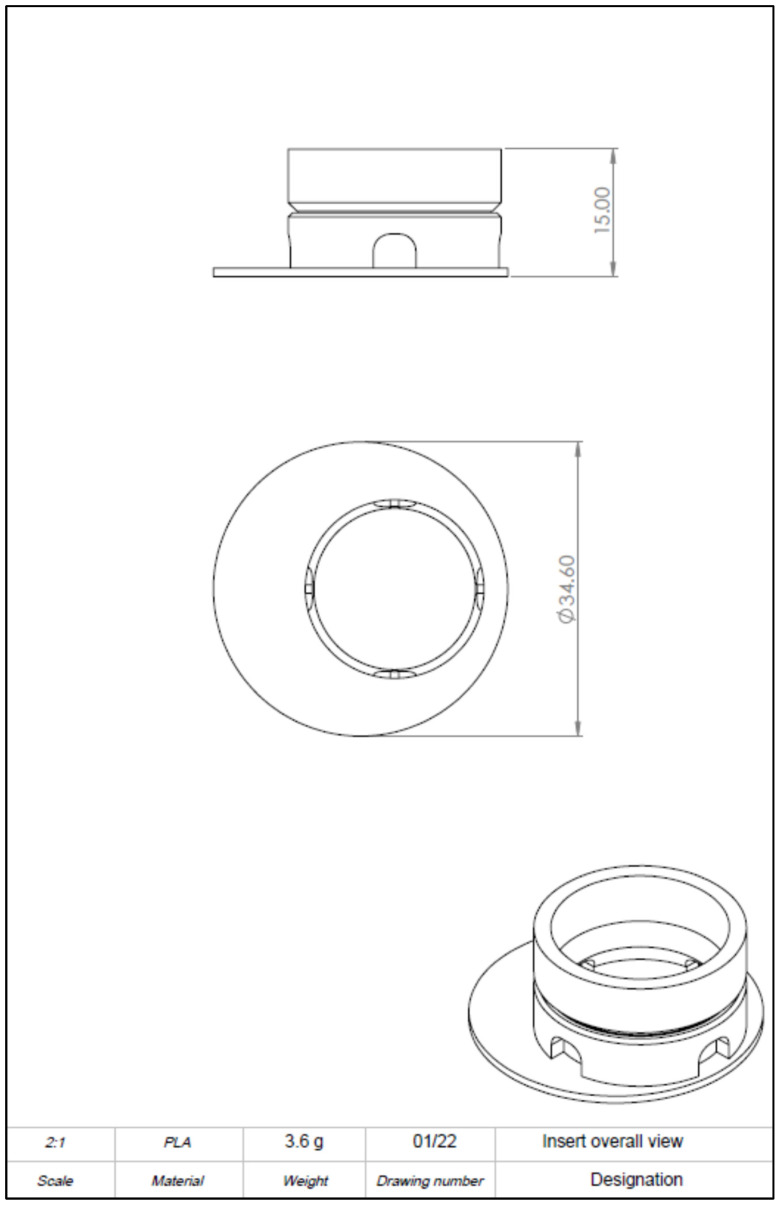
Drawing derivation of computer-aided design (CAD) model of the 3D-printed cell culture insert showing dimension and oblique crack.

**Figure A3:**
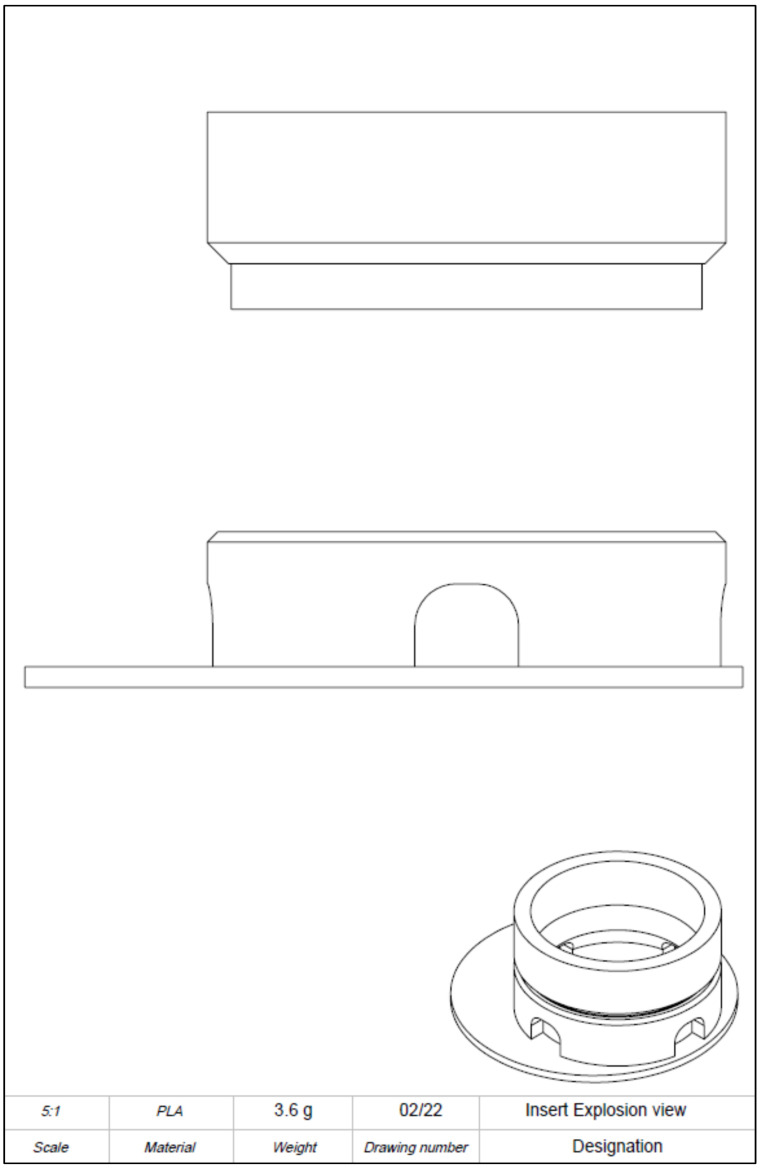
Drawing derivation of computer-aided design (CAD) model of the 3D-printed cell culture insert. Detailed view.

**Figure A4:**
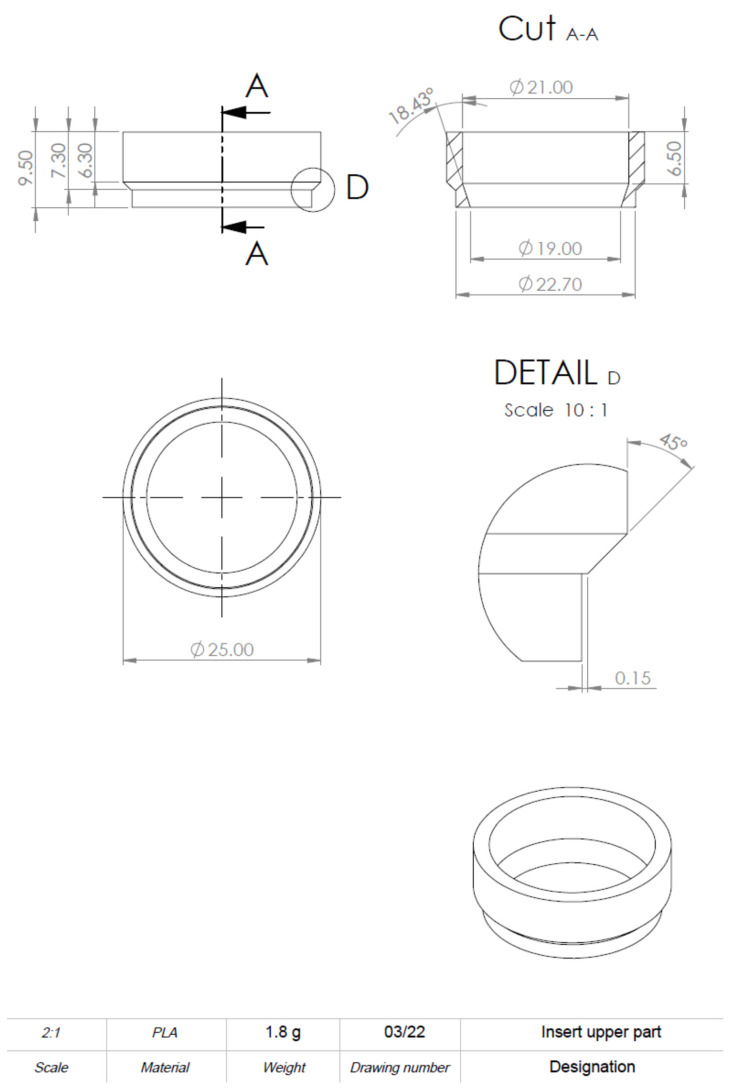
Drawing derivation of computer-aided design (CAD) model of the 3D-printed cell culture insert. Detailed information on dimensions.

**Figure A5:**
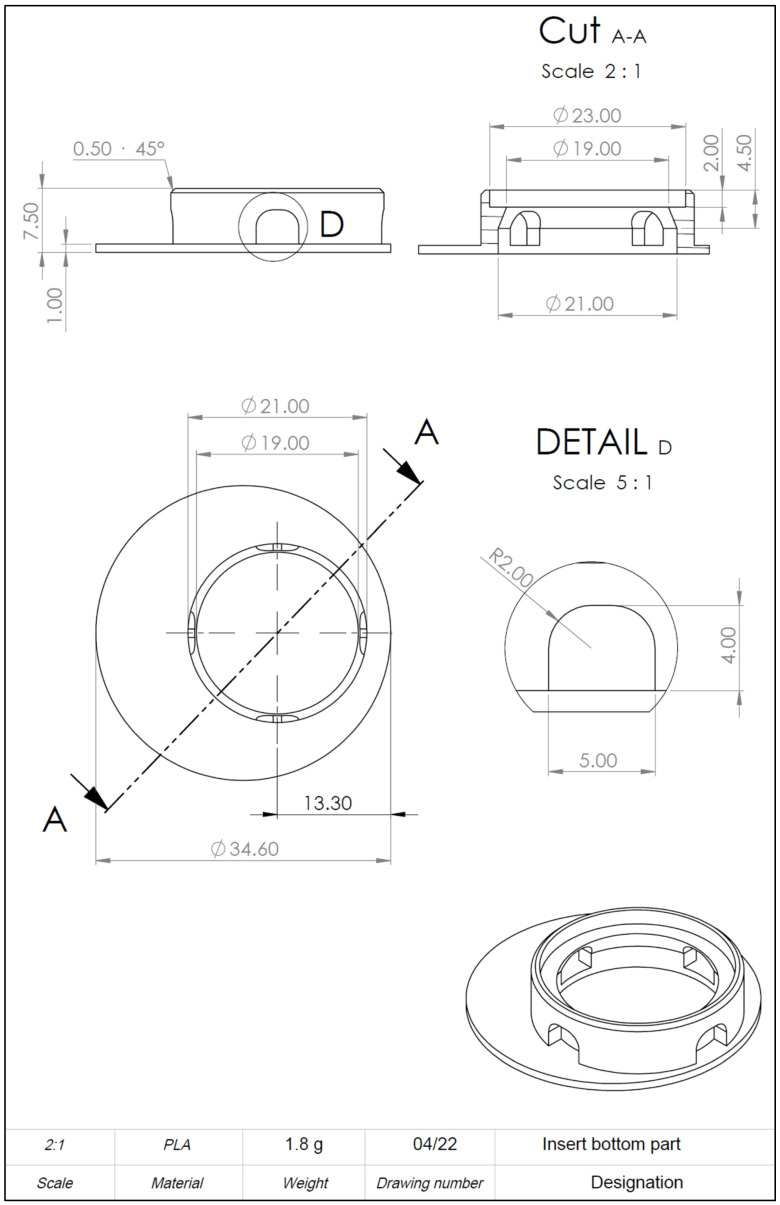
Drawing derivation of computer-aided design (CAD) model of the 3D-printed cell culture insert. Detailed information on dimensions.

## Data Availability

The data presented in this study are available on request from the corresponding author.

## Supplementary Materials

The following are available online at https://www.mdpi.com/article/10.3390/life12081216/s1, Construction templates S1: insert_upper_part.stl, S2: insert_bottom_part.stl, Video S1: Video insert in well plate.avi.
